# Survival and Lung Function Changes in Hypersensitivity Pneumonitis According to Radiological Phenotypes Compared With Idiopathic Pulmonary Fibrosis

**DOI:** 10.7759/cureus.57307

**Published:** 2024-03-31

**Authors:** Gabriel Juliá-Serdá, Javier Navarro-Esteva, Laura Doreste-Salgado, Ibrahim Véliz-Flores, Rubén Pestana-Santana, Jesús María González-Martín, Felipe Rodríguez-de Castro

**Affiliations:** 1 Pulmonary Medicine, Hospital Perpetuo Socorro, Las Palmas de Gran Canaria, ESP; 2 Pulmonary Medicine, Hospital Universitario de Gran Canaria Doctor Negrín, Las Palmas de Gran Canaria, ESP; 3 Radiodiagnosis, Hospital Universitario de Gran Canaria Doctor Negrín, Las Palmas de Gran Canaria, ESP; 4 Biostatistics, Hospital Universitario de Gran Canaria Doctor Negrín, Las Palmas de Gran Canaria, ESP

**Keywords:** survival, computed tomography, idiopathic pulmonary fibrosis, hypersensitivity pneumonitis, interstitial lung diseases

## Abstract

Introduction: The main objective of this study was to estimate survival and changes in lung function in patients with chronic hypersensitivity pneumonitis (HP), both fibrotic (f-HP) and nonfibrotic (nf-HP), and to compare them with those in patients with idiopathic pulmonary fibrosis (IPF).

Methods: HP was diagnosed based on antigen exposure, HRCT (high-resolution CT scan), BAL (bronchoalveolar lavage), and histology. According to HRCT, HP was classified into fibrotic and non-fibrotic phenotypes. In most cases, IPF was diagnosed based on HRCT findings.

Results: We identified 84 patients: 46 with IPF, 18 with f-HP, and 20 with nf-HP. Five-year survival was 23.9% in IPF, 72% in f-HP, and 100% in nf-HP (p <0.0001). Honeycombing was associated with decreased survival in IPF (p <0.001) and in f-HP (p <0.0001). The mean loss of FVC (forced vital capacity) % pred. (percent predicted) was -18.3% in IPF (p =0.001), -4.8% in f-HP, and -6.0% in nf-HP. The mean change in DLCO (diffusion capacity for carbon monoxide) % pred. was -10.2% in IPF (p <0.002), -0.5% in f-HP, and +1.9% in nf-HP. The agreement between radiological phenotypes and histology in HP was 89.6%.

Conclusions: We found shorter survival in IPF, followed by f-HP, and nf-HP. Over time, we did not find significant changes in FVC% pred. or DLCO% pred. in HP, while a significant decline in IPF was noted. In HP, we found strong agreement between radiological phenotypes and histology. Radiological signs suggestive of lung fibrosis in HP were reliable for the diagnosis of f-HP and seem to have intrinsic prognostic value.

## Introduction

Hypersensitivity pneumonitis (HP) is an immunologically induced lung disorder resulting from repeated exposure to a wide variety of inhaled antigens [1,2]. HP patients display a large heterogeneity of clinical presentations and outcomes, with subtypes historically categorized by disease duration at the time of presentation (i.e., acute, subacute, or chronic) [2-4]. However, these categories are vaguely defined in the existing literature and are not consistently associated with post-diagnosis survival [5]. While avoidance of the responsible antigen can lead to complete recovery in most patients with acute HP, others show loss of pulmonary function and develop progressive fibrosis and respiratory failure, irrespective of having acute, subacute, or chronic HP [1,5,6]. Accordingly, the current international guidelines recommend that patients should be classified as fibrotic HP (f-HP) that can hardly be distinguished from idiopathic pulmonary fibrosis (IPF) [7], or non-fibrotic HP (nf-HP), as determined by the presence or absence of radiological and/or histopathological fibrosis [5,6]. Previous studies have not always considered the radiologic and histologic pattern of fibrosis when assessing these patients [8-10]. In addition, there are few research reports, sometimes with mixed results, comparing survival and changes in lung function in chronic HP and IPF patients [11,12]. Our objectives were: (1) to assess survival and changes in forced vital capacity (FVC% pred.) and carbon monoxide transfer test (DLCO% pred. (diffusion capacity for carbon monoxide percent predicted)) in HP patients, based on the presence or absence of radiological findings of fibrosis (f-HP vs. nf-HP) on high-resolution CT scan (HRCT), and compare them to subjects with IPF, and (2) to estimate the agreement between radiological phenotype and histology in HP.

## Materials and methods

Diagnostic criteria

This was a single-center observational study conducted at Hospital Universitario de Gran Canaria “Dr. Negrín”, a reference center for emergencies and admissions for 380,000 adult inhabitants in Las Palmas de Gran Canaria, Spain. We retrospectively analyzed two cohorts of patients.

Patients Diagnosed With IPF Between 2001 and 2022

Before the year 2011, we exclusively included cases (n=9) with a definitive diagnosis of IPF (subjects with histological findings of usual interstitial pneumonia (UIP) on lung biopsy obtained by video-assisted thoracoscopic surgery (VATS), after other known causes of this histologic pattern were ruled out). These patients had an interstitial lung disease in which the cause remained elusive after an extensive diagnostic workup and, in a clinical session, a surgical lung biopsy (SLB) was proposed. After 2011, a Multidisciplinary Committee on Vascular and Interstitial Lung Diseases diagnosed cases (n=37) through consensus, based on the international guidelines for the diagnosis of IPF [7]. The clinical characteristics, the Charlson comorbidity index, a complete blood count, serum chemistry, antinuclear antibodies, chest X-ray, HRCT, spirometry, DLCO (single breath), and bronchoalveolar lavage (BAL) cell counts were collected.

Patients With a Definite Diagnosis of HP Between 2001 and 2022

**Figure 1:**
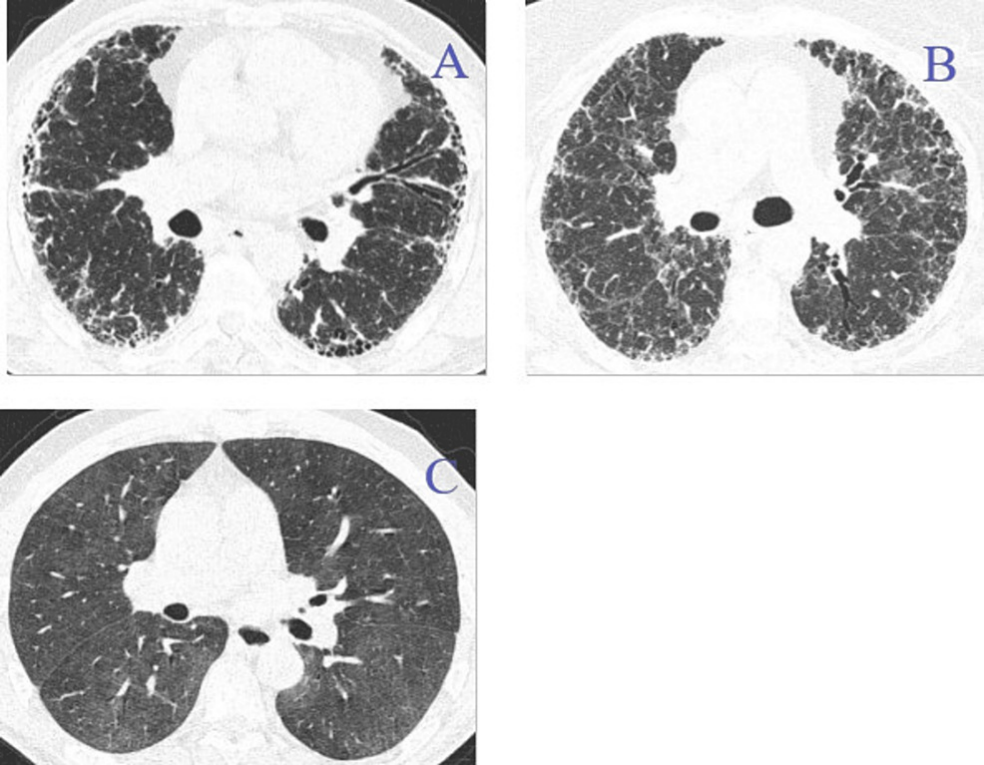
Tomographic signs of IPF (idiopathic pulmonary fibrosis), f-HP (fibrotic hypersensitivity pneumonitis), and nf-HP (nonfibrotic hypersensitivity pneumonitis). (A) IPF, probable pattern: peripheral reticulation, traction bronchiectasis, and honeycombing. (B) F-HP: reticulation, traction bronchiectasis, and ground glass opacities. No honeycombing was noted in this case. (C) Nf-HP: diffuse ground glass opacities. There are no indications of lung fibrosis.

The majority of HP patients were diagnosed with lung biopsy by VATS (n=29). In cases without surgical biopsy (n=9), especially in recent years, the diagnosis was established when all the following criteria were fulfilled: known causal agent, compatible HRCT findings, BAL cell count with more than 25% lymphocytes, and presence of non-necrotizing granulomas in the transbronchial biopsy or cryobiopsy. In the latter group, two patients were diagnosed when the first three criteria previously mentioned were met. According to HRCT findings, two phenotypes of HP were identified: (1) f-HP phenotype: presence of honeycombing and/or the combination of traction bronchiectasis and reticular pattern and (2) inflammatory or nf-HP phenotype: absence of previous criteria along with the presence of ground glass imaging, predominantly in the upper lobes, with or without centrilobular micronodules, mosaic pattern, and air trapping (when expiratory images were available). Patients with either isolated reticulation or traction bronchiectasis were included in this phenotype. The presence of a mosaic pattern that was accentuated on expiratory images was also recorded. A suggestive HRCT image for every condition is shown in Figure 1.

Those who underwent SLB were classified as either f-HP or nf-HP as follows: (1) fibrotic pattern: either airway-centered interstitial fibrosis, fibrotic nonspecific interstitial pneumonia (f-NSIP) pattern, or usual interstitial pneumonia (UIP) pattern; (2) non-fibrotic (cellular) pattern: bronchiolocentric lymphocytic interstitial pneumonitis with chronic bronchiolitis, and small and poorly constituted non-necrotizing granulomas [1]. In addition to a detailed medical history, the Charlson comorbidity index, a complete blood count, serum chemistry, serum-specific IgG against various antigens according to data on epidemiological exposure, antinuclear antibodies, chest X-ray, HRCT, spirometry, DLCO, bronchoscopy with BAL, and/or transbronchial biopsy were also collected.

IPF and HP patients were followed up from 1 to 252 months, with a median of 62.5 months. If applicable, the date of lung transplantation or death was registered. Follow-up data were obtained from the electronic records for each patient. In cases diagnosed before the implementation of the computerized medical record system, individual files were reviewed. The study was approved by the Hospital's Research Ethics Committee.

Statistical analysis

Data are expressed as absolute values and their frequency and corresponding percentage for qualitative variables. The mean, standard deviation, median, 25th, and 75th percentiles were calculated for the quantitative variables. The Kolmogorov-Smirnov test was used to check for normality. The Mann-Whitney U test was employed to compare two continuous variables, and the Kruskal-Wallis tests were used to compare more than two continuous variables. The exact Fisher test was carried out to compare categorical variables. The Kaplan-Meier method was used to construct survival curves, and the Log-rank test was used to compare curves. To calculate the estimated survival in each cohort, the median was considered. A p-value less than 0.05 was considered significant. Hypotheses contrasts were calculated with two tails. The statistical program used was R Core Team 2021, version 4.1.2.

## Results

Patient characteristics

A total of 84 patients were evaluated: 46 with IPF, 18 with f-HP, and 20 with nf-HP. Patient demographics and clinical features at the time of diagnosis are presented in Table 1. All patients reported experiencing dyspnea for at least six months. In HP, at least one causal agent was identified in 26 out of 38 cases (68.4%): 9 out of 18 cases in f-HP and 17 out of 20 cases in nf-HP (p < 0.05).

**Table 1:** Demographics and other variables in IPF, f-HP, and nf-HP patients *Comparing IPF with f-HP and nf-HP. **For all comparisons. ***Comparing IPF with nf-HP. ^¶^Comparing IPF with f-HP. #Some patients had more than one antigen exposure. N: number; SD: standard deviation; M: male; F: female; N/A: not applicable; NS: non-significant; FVC: forced vital capacity; LBA: lung bronchoalveolar; DLCO: diffusion capacity for carbon monoxide; IPF: idiopathic pulmonary fibrosis;  f-HP: fibrotic hypersensitivity pneumonitis; nf-HP: nonfibrotic hypersensitivity pneumonitis.

Variable	IPF	F-HP	Nf-HP	p-value
Patients, n	46	18	20	
Sex, n (%)	M: 39 (84.7)	M: 2 (11.1)	M: 6 (30)	p<0.001*
	F: 7 (15.2)	F: 16 (88.8)	F: 14 (70)	P<0.001*
Age, n (SD)	68.6 (8.8)	61.1 (8.5)	47.4 (9.6)	p<0.05**
Tobacco smoking				
Active, n (%)	5 (10.9)	3 (16.7)	3 (15)	
Ex-smoker, n (%)	31 (67)	3 (16.7)	3 (15)	P<0.001*
Non-smoker, n (%)	10 (21.7)	12 (66.7)	14 (70)	
Charlson Comorbidity Index (SD)	2.35 (1.34)	1.67 (0.91)	1.35 (0.59)	p=0.004***
Antigen exposure^#^	N/A			
A (avian), n		2	10	
M (molds, air conditioning), n		10	9	
P (animal proteins), n		1	3	
O (others), n		4	4	
U (unknown exposure), n		9	3	
Squeaks, n (%)	2 (4.3%)	5 (27.7%)	4 (20%)	NS
Finger clubbing	10 (21.7%)	2 (11.1%)	1 (5%)	p=0.017*
FVC baseline % (SD)	81.8 (20.3)	68.2 (12.2)	69.5 (15.6)	p=0.021^¶^
DLCO baseline % (SD)	43.9 (16.3)	32.7 (12.7)	42.5 (17.4)	NS
LBA lymphocytes % (SD) (n)	4.5 (5.8) (23)	37.1 (21.5) (16)	39.1 (22.1) (18)	p<0.001*
LBA lymphocytosis > 25%, n (%)	N/A	11/16 (68.7)	13/18 (72.2)	NS
Antinuclear antibodies ≥ 1:160, n (%)	14 (30.4)	4 (22.2)	3 (15)	p<0.01*
HRCT findings				
Honeycombing, n	35	4	0	
Traction bronchiectasis, n	42	16	0	
Interlobular septal thick, n	29	10	4	
Subpleural predominance, n	20	0	0	
Ground glass, n	14	9	8	
Random nodules, n	0	4	7	
Mosaic pattern, n	0	7	10	
Surgical biopsy, n (%)	13 (28.2)	16 (88.8)	14 (70)	NS
Radiological and histological concordance, n (%)	N/A	15/16 (93.7)	12/14 (85.7)	NS

BAL cellular analysis

The mean percentage of macrophages was 86.3 ± 11.5% in IPF, 50.5 ± 18.1% in f-HP, and 50.1 ± 21.3% in nf-HP (p < 0.001 for IPF versus HP as a group). The mean percentage of lymphocytes was 4.5 ± 5.8% in IPF, 37.1 ± 21.5% in f-HP, and 39.1 ± 22.1% in nf-HP (p < 0.001 for IPF versus HP as a group). There were no significant differences in the percentages of the remaining cells. The mean CD4/CD8 ratio was calculated for the two HP groups, and no significant differences were found, with a mean ratio of 4.8 ± 4.5 in f-HP and a mean ratio of 3 ± 2.7 in nf-HP.

Lung function

**Figure 2:**
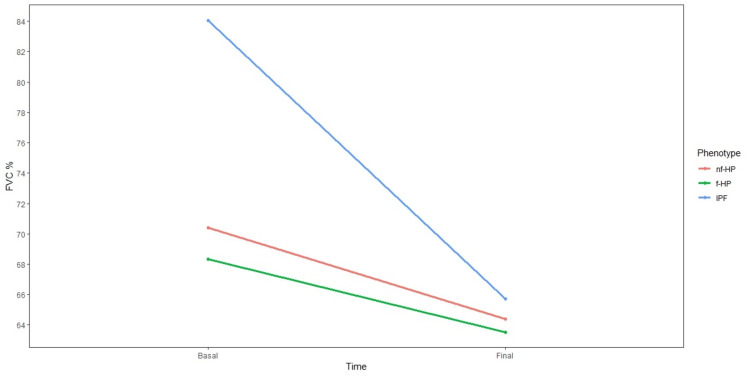
FVC % pred. (percent predicted) trends for the three cohorts. The fall in FVC % pred. was more pronounced in IPF as opposed to f-HP or nf-HP. FVC: forced vital capacity; IPF: idiopathic pulmonary fibrosis; f-HP: fibrotic hypersensitivity pneumonitis; nf-HP: non-fibrotic hypersensitivity pneumonitis.

A total of 398 FVC measurements were available, with an average of 3.7 measurements per subject. Forty-one subjects with IPF, 15 with f-HP, and 19 with nf-HP had at least two FVC measurements. The decline in FVC% pred. was significantly more pronounced in IPF compared to f-HP or nf-HP (p = 0.009) (Figure 2). The last measurement obtained from patients with IPF showed a mean FVC% pred. of 65.7 ± 20.2%, representing a loss of -18.3% (95% CI -13.3 to -23.3%, p < 0.001) compared to baseline values. Those with f-HP and nf-HP had a mean final FVC% pred. of 63.5 ± 12.4% and 64.3 ± 21.5%, representing a loss of -4.8% and -6.0%, respectively. There was no significant difference between the decline in FVC% pred. in f-HP compared to nf-HP. Regarding the FEV1/FVC ratio in HP, there was not a single case with a result of less than 0.7, ruling out significant airflow obstruction.

A total of 297 DLCO tests were available, with an average of 3.5 measurements per subject (range 0-6). Thirty-one subjects with IPF, 14 with f-HP, and 19 with nf-HP had at least two DLCO assessments. The decline in DLCO% pred. was significantly more pronounced in IPF compared to nf-HP (p = 0.033). At the final measurement, patients with IPF had a mean DLCO% pred. of 34.9 ± 15.2%, representing a loss of -10.2% compared to baseline values. Those with f-HP and nf-HP had a mean final DLCO% pred. of 30.2 ± 10.8% and 43.3 ± 22.5%, representing a loss of -0.5% and a gain of 1.9%, respectively. The difference in the final DLCO% pred. between f-HP and nf-HP was -13.1% (95% CI -24.1 to -2.1%, p = 0.02).

Radiological phenotype and histological correlation in HP

To reach a definitive diagnosis, an SLB by VATS was performed in 29 out of 38 patients with suspected HP. In line with the recommendations provided by the ATS/JRS/ALAT Clinical Practice Guidelines [1], a pathological diagnosis of nf-HP was confirmed with confidence in 13 out of the 14 (93.3%) patients who underwent SLB. Only one case lacked poorly formed granulomas, resulting in a nondefinite diagnosis. As for f-HP, the histological pattern was compatible with this diagnosis in 13 out of 15 (88.2%) cases. Regarding the correlation between radiological phenotype and histological pattern, an agreement was found in 26 out of 29 (89.6%) cases (Cohen’s kappa coefficient = 0.8). Of the three cases with diagnostic disagreement, two were classified as non-fibrotic based on histology and fibrotic on HRCT, and the third as fibrotic on histological criteria and non-fibrotic on radiological criteria.

Survival analysis

**Figure 3:**
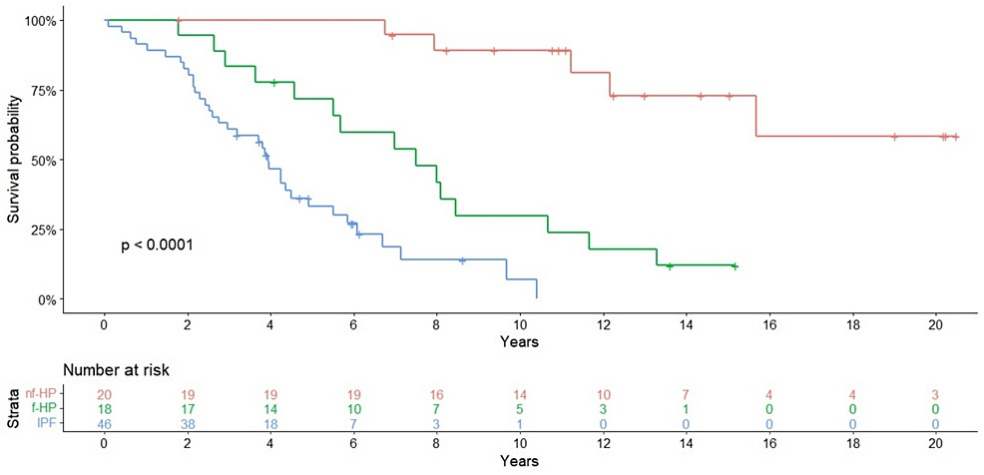
Kaplan-Meier curves of overall survival probability for the studied cohorts. Nf-HP: non-fibrotic hypersensitivity pneumonitis; f-HP: fibrotic hypersensitivity pneumonitis; IPF: idiopathic pulmonary fibrosis.

The number of primary events in our series, understood as death or transplantation, was 36 (78.2%) (2 transplanted, 34 deceased) in patients with IPF, 14 (77.7%) in patients with f-HP, and 5 (25%) in patients with nf-HP. To calculate the estimated survival in each cohort, the median was considered. In IPF, the median survival was 3.95 years, with 91.3% of patients alive in the first year after diagnosis and 23.9% alive at five years. The median survival in f-HP patients was 7.5 years, with a survival of 100% in the first year after diagnosis and 72% at five years. All nf-HP patients were alive at five years after diagnosis. Significant differences in five-year survival were observed in IPF versus nf-HP groups (HR: 20.6, 95% CI 6.7-63.5; p < 0.001); and in f-HP versus nf-HP patients (HR: 7.2, 95% CI 2.4-22.0; p < 0.001) (Figure 3).

**Figure 4:**
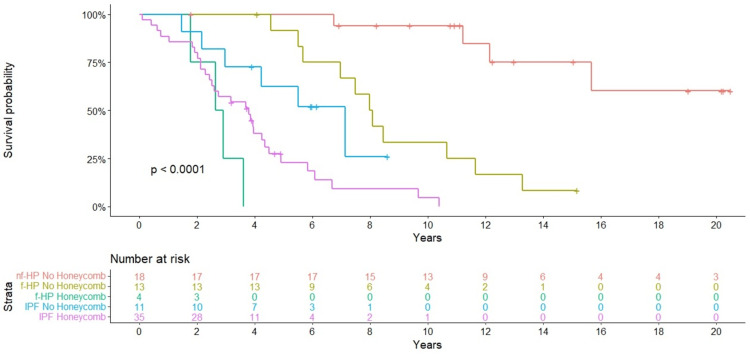
Kaplan-Meier survival curves for the three cohorts according to the presence of honeycomb changes in HRCT. Nf-HP: non-fibrotic hypersensitivity pneumonitis; f-HP: fibrotic hypersensitivity pneumonitis; IPF: idiopathic pulmonary fibrosis; HRCT: high-resolution CT scan.

When mortality was evaluated according to the presence of honeycomb changes in HRCT, a significant difference was found in IPF (HR = 43.8, 95% CI 11.5-165.6, p < 0.01) and f-HP (HR = 106.1, 95% CI 19.7-570.0, p < 0.0001) (Figure 4). When survival was compared according to the degree of BAL lymphocytosis (≥ 25% versus < 25%) in HP patients, no significant difference was found. Similarly, survival in IPF was not affected by the presence of serum ANA titers ≥ 1:160.

Thirty-six (78.2%) IPF patients were treated with antifibrotics, of whom 19 (52.8%) received pirfenidone, 11 (30.5%) nintedanib, and 6 (16.6%) both drugs, administered sequentially. All untreated patients (n=10) suffered a primary event (death or transplantation), at an average of 1.93 years, and showed a five-year survival of 10%. Twelve patients (37.5%) who received antifibrotics experienced the primary event, resulting in a median survival of 3.96 years and a survival rate of 27.7% at five years. Although a significant difference between these groups was not observed, survival declined more rapidly in the untreated patients during the first three years after diagnosis.

## Discussion

We have studied a cohort of patients with IPF and HP diagnosed by strict criteria. Among the subjects with HP, we have defined two mutually exclusive HRCT phenotypes, fibrotic and non-fibrotic, and observed a high degree of agreement between this two-pattern radiological description and the histopathological stage of the disease at the time of diagnosis. Patients with IPF were significantly older, as IPF is usually diagnosed after 60 years of age, and HP in younger subjects [11,12]. We found that subjects with f-HP are older, have higher unidentified exposure, and lower BAL lymphocytes, FVC% pred., and DLCO% pred., compared to nf-HP patients [1,5]. Several studies have shown that the presence of histopathological features of fibrosis is associated with a poor prognosis, but previous publications do not always include the pattern of radiologic fibrosis in their analyses [8-10]. The most relevant finding of our work is that the two radiological phenotypes of HP are associated with different likelihoods of survival. Patients with nf-HP had significantly longer survival than those with f-HP, and this latter group seems to fare better than patients with IPF. The fact that HP patients without signs of fibrosis have a better prognosis is consistent with what is described in the literature, so our study supports the idea of differentiating HP patients based on fibrotic features detected by HRCT [9-13]. Mooney et al. [14] found that chronic HP had better survival than IPF. However, Alberti et al. [15] observed that chronic HP had a similar survival time when compared with IPF. Their HP patients were older than ours (62.7 years vs. 54.2 years), while the number of cases with a histological diagnosis is larger in our series. Comparatively, we were able to confirm the diagnosis with a higher level of certainty. The findings of Salisbury et al. [16] in nf-HP and f-HP are similar to ours. They defined a third radiological HP phenotype with honeycomb predominance without differences in survival compared with IPF. Ojanguren et al. also found that honeycomb changes were associated with shortened survival in HP [17]. Lastly, regarding the association between mosaic attenuation and survival, we did not find a favorable correlation as described by Chung et al. [18].

The previous classification of HP into acute, subacute, and chronic relied on the duration of the symptoms and provided limited data for therapeutic and prognostic estimations [4,13,19]. Our results suggest that the objective assessment of lung fibrosis by HRCT is a useful predictor of survival and could help make better clinical decisions. These findings are in line with the recommendations published by the ATS, JRS, and ALAT Societies [1].

Concerning pulmonary function, patients with IPF presented surprisingly higher baseline FVC% pred. and DLCO% pred. values than those with f-HP and nf-HP, although the IPF group experienced a greater decline thereafter. IPF usually behaves more aggressively, so it is likely that patients are more aware of symptoms earlier in the course of the disease and seek medical help at a time when lung function is not severely impaired. This would contrast with a relatively more indolent course in f-HP patients. The faster decline in lung function observed in IPF compared to f-HP may also explain the shorter survival observed in IPF patients. The type of interstitial lung disease (IPF vs. f-HP vs. nf-HP) and decline in DLCO% pred. were independently associated with worse survival. Regarding DLCO, in their multivariate analysis, Ojanguren et al. [17] and Macaluso et al. [20] described similar findings. However, other studies did not include DLCO% pred. in their final adjusted model but showed that FVC% pred. was a better predictor of survival [15,16]. Finally, patients with nf-HP experienced an improvement in DLCO% pred., possibly related to antigen avoidance and/or treatment with oral steroids.

Concerning the cellular pattern found in the BAL, the most relevant aspect of our study was the presence of a high percentage of lymphocytes in f-HP and nf-HP, with significant differences compared to IPF. A high number of lymphocytes in the BAL is a frequent finding in HP, consistent with the immunological dysregulation described in its pathogenesis [3]. It is relevant to note that BAL lymphocytosis may help differentiate f-HP from IPF [21]. We found a non-inverted mean CD4/CD8 ratio in f-HP and nf-HP; however, there is increasing evidence that this ratio does not have intrinsic value and should not be used for diagnostic purposes [22]. Finally, a moderate increase in neutrophils and eosinophils has been described in patients with IPF, but this was not consistent with our findings [23].

Almost 80% of our IPF patients received either nintedanib or pirfenidone. We observed early differences in survival that leveled off around the fourth year after diagnosis. Previous studies have also shown the beneficial effect of these drugs in slowing lung function decline, which justifies their use in our healthcare system since 2014 [24,25].

Our study has several limitations. First, there is not a universal diagnostic algorithm for HP. Therefore, comparing our findings with those described in the literature could pose difficulties. In this regard, our diagnostic criteria have been very strict, with histological confirmation in most cases. Second, this is a relatively small sample from a single center. Therefore, the statistical power of the study may be limited. Third, the retrospective nature has inherent limitations, such as potential selection bias and the limited value of the gathered survival data. Finally, the data were collected from medical records, so their validity depends on their reliability. Despite these limitations, we believe that these results can be extrapolated and contribute to the current knowledge of these diseases.

## Conclusions

Based on a high level of diagnostic confidence, our study suggests that the described radiological phenotypes may help stratify patients with HP into f-HP and nf-HP, with added prognostic value. We found that patients with nf-HP experience longer survival, followed by those with f-HP, and finally, those with IPF. The presence of radiological honeycombing at diagnosis seems to be a risk factor for decreased survival in both f-HP and IPF. The trends in FVC% pred. and DLCO% pred. were unfavorable in IPF, whereas changes were less significant in f-HP and nf-HP.
